# Vertical transmission explains the specific *Burkholderia* pattern in *Sphagnum* mosses at multi-geographic scale

**DOI:** 10.3389/fmicb.2013.00394

**Published:** 2013-12-18

**Authors:** Anastasia Bragina, Massimiliano Cardinale, Christian Berg, Gabriele Berg

**Affiliations:** ^1^Institute of Environmental Biotechnology, Graz University of TechnologyGraz, Austria; ^2^Institute of Plant Sciences, Karl-Franzens University of GrazGraz, Austria

**Keywords:** *Sphagnum fallax*, *Sphagnum magellanicum*, *Burkholderia* communities, amplicon pyrosequencing, FISH-CLSM

## Abstract

The betaproteobacterial genus *Burkholderia* is known for its versatile interactions with its hosts that can range from beneficial to pathogenic. A plant-beneficial-environmental (PBE) *Burkholderia* cluster was recently separated from the pathogen cluster, yet still little is known about burkholderial diversity, distribution, colonization, and transmission patterns on plants. In our study, we applied a combination of high-throughput molecular and microscopic methods to examine the aforementioned factors for *Burkholderia* communities associated with *Sphagnum* mosses – model plants for long-term associations – in Austrian and Russian bogs. Analysis of 16S rRNA gene amplicons libraries revealed that most of the *Burkholderia* are part of the PBE group, but a minor fraction was closely related to *B. glathei* and *B. andropogonis* from the pathogen cluster. Notably, *Burkholderia* showed highly similar composition patterns for each moss species independent of the geographic region, and *Burkholderia*-specific fluorescent *in situ* hybridization of *Sphagnum* gametophytes exhibited similar colonization patterns in different *Sphagnum* species at multi-geographic scales. To explain these patterns, we compared the compositions of the surrounding water, gametophyte-, and sporophyte-associated microbiome at genus level and discovered that *Burkholderia* were present in the *Sphagnum* sporophyte and gametophyte, but were absent in the flark water. Therefore, *Burkholderia* is a part of the core microbiome transmitted from the moss sporophyte to the gametophyte. This suggests a vertical transmission of *Burkholderia* strains, and thus underlines their importance for the plants themselves.

## Introduction

The genus *Burkholderia*, which was described by Yabuuchi et al. (1992), encompasses a diverse group of Betaproteobacteria with currently more than 60 validly described species. *Burkholderia* species are known for their beneficial as well as pathogenic interaction with plants, animals, and humans (Coenye and Vandamme, 2003). In the past, most studies focused on the pathogenic species for their enormous clinical importance (Mahenthiralingam et al., 2005). Recently, a specific plant-beneficial-environmental (PBE) *Burkholderia* cluster that contains non-pathogenic species was divided from the cluster that comprises human, animal and plant pathogens (Caballero-Mellado et al., 2007; Suárez-Moreno et al., 2010, 2012). However, there is no clear border between both groups especially within the plant-associated species; for example *B. glathei* was suggested to be transfered from the pathogenic to the PBE group (Verstraete et al., 2013). Many PBE members belong to *Burkholderia* species symbiotic to tropical plants; each nodulating plant species is colonized by a single unique endophytic *Burkholderia* species (Van Oevelen et al., 2002; Lemaire et al., 2011). Several species from the PBE cluster share characteristics that are of use in association with plants, such as quorum sensing systems, the presence of nitrogen fixation and/or nodulation genes, and the ability to degrade aromatic compounds (Suárez-Moreno et al., 2012), and many of them are characterized by an endophytic lifestyle (Sessitsch et al., 2005; Gasser et al., 2009; Mitter et al., 2013). While single strains of the PBE cluster are already well-characterized, little is known about the ecology and colonization pattern of *Burkholderia* species on plants.

Plants have been recognized as meta-organisms due to their close symbiotic relationship with their microbiome that fulfills important host functions (Berg, 2009; Bulgarelli et al., 2012; Hirsch and Mauchline, 2012; Lundberg et al., 2012; Berg et al., 2013). These advances were driven by both “omic”-technologies guided by next-generation sequencing (NGS) and microscopic insights (Berendsen et al., 2012; Jansson et al., 2012). Mosses belong to the phylogenetically oldest group of land plants on Earth, and their long-term intense relationship with their associated microbes has contributed to the co-evolution of a highly specific microbiome (Opelt and Berg, 2004; Opelt et al., 2007c; Bragina et al., 2012). Therefore, mosses are important models in studying plant-microbe interactions and the ecology of plant-associated bacteria. The genus *Sphagnum* is among the most abundant and cosmopolitan of bog vegetation in the Northern hemisphere, and greatly contributes to both global carbon turnover and global climate (Raghoebarsing et al., 2005; Dise, 2009). The ecological significance of bogs is directly related to the physical, morphological, and chemical characteristics of *Sphagnum* peat mosses which set *Sphagnum* apart from other mosses in practically every stage of the life cycle (Daniels and Eddy, 1985). *Burkholderia* species play an important role for *Sphagnum* mosses and peatland ecosystem (Opelt et al., 2007a,b), and new *Burkholderia* species, which belong to the PBE cluster, have recently been isolated from these mosses (Vandamme et al., 2007). However, their composition and occurrence on *Sphagnum* at various geographical scales—ranging from the moss gametophyte and sporophyte up to continental level—is not yet understood. We hypothesize that *Sphagnum* species are colonized by specific *Burkholderia* from the PBE cluster independent from the geographic region.

To study this hypothesis and understand the ecological role, composition, colonization, as well as distribution pattern on plants, we studied *Burkholderia* communities on two *Sphagnum* species (*S. magellanicum* and *S. fallax*) associated with different a-biotic parameters from different bogs in Austria and Russia. We used an assortment of methods combining the analysis of *Burkholderia*-specific 16S rRNA gene pyrosequencing libraries with FISH-CLSM analysis. Furthermore, we compared the compositions of water, gametophyte-, and sporophyte-associated microbiomes to understand the transmission and distribution patterns of the *Burkholderia* communities.

## Materials and methods

### Sampling design

To analyze the diversity and distribution pattern of the *Sphagnum*-associated *Burkholderia* community, *S. magellanicum* BRID. (section *Sphagnum*) and *S. fallax* H. KLINGGR. (section *Cuspidata*) were selected. Both bryophytes are members of the typical and cosmopolitan vegetation in peat bogs (Daniels and Eddy, 1985). Adult gametophytes of mosses were sampled in three acidic peat bogs in Austria and in three acidic peat bogs in Russia in September 2009 and July 2010, respectively (Table S1). Four single replicates per *Sphagnum* species (15–20 plantlets) were collected in each of the investigated bogs at a minimum distance of about 40 m from each other. The plant samples were placed into sterile plastic bags and transported to the laboratory. In addition, two sporophyte samples of *S. fallax* consisting of enclosed spore capsules, and one water sample from a small wet depression (flark) were collected into sterile screw cap tubes and processed separately.

### Total-community DNA isolation

The microbial fractions associated with moss gametophytes and sporophytes were extracted as previously described (Bragina et al., 2012). In short, 5 g of plant material were physically disrupted and resuspended in 10 ml of 0.85% NaCl. 2 ml of suspension were centrifuged at 13,000 r.p.m. for 20 min at 4°C and the supernatant was discarded. For extraction of the sporophyte-associated microbial community, 10 enclosed spore capsules per sample were surface-sterilized and ground with 1.5 ml of 0.85% NaCl. The ground suspension was centrifuged at 13,000 r.p.m. for 20 min at 4°C and the supernatant was discarded. The pellet from the flark water sample was obtained through several rounds of centrifugation at 10,000 r.p.m. for 15 min at 4°C until a constant pellet size was obtained. The resulting cell pellets were applied for isolation of the total-community DNA using the FastDNA® SPIN Kit for Soil (MP Biomedicals, Solon, OH, USA). Final aliquots of the total-community DNA were further used for a deep sequencing-approach.

### 454-pyrosequencing and bioinformatic processing

The diversity of the *Sphagnum*-associated microbiome with a special focus on the genus *Burkholderia* was investigated using a barcoded pyrosequencing technology. For this purpose, 16S rDNA amplicons were generated using Taq-&Go™ Ready-to-use PCR Mix (MP Biomedicals, Solon, OH, USA). The total-community DNA of gametophyte samples was selectively amplified with *Burkholderia*-specific primers BKH143Fw/BKH1434Rw (Schönmann et al., 2009) followed by amplification with universal bacterial primers Unibac-II-515f/Unibac-II-927r (Lieber et al., 2003). In addition, the total-community DNA of *S. fallax* gametophyte samples from the bog Pürgschachen Moor (Table S1) and flark water sample was amplified with universal bacterial primers Unibac-II-515f/Unibac-II-927r. The total-community DNA of sporophyte samples was amplified with universal bacterial primers 799f/1492r (Lane, 1991; Chelius and Triplett, 2001) because application of the Unibac-II-515f/Unibac-II-927r achieved mostly plant-derived sequences (data of preliminary experiments). Primer sequences are listed in Table 1. Duplicate PCR products from all templates were purified with Wizard® SV Gel and PCR Clean-Up System (Promega, Madison, WI, USA). Amplicons derived from the same *Sphagnum* sp. and sampling site were pooled in equimolar ratios and subjected to pyrosequencing using the Roche 454 GS FLX and FLX+ Titanium platforms performed by LGC Genomics (Berlin, Germany) and Eurofins MWG (Ebersberg, Germany), respectively. In total, we produced 12 pyrosequencing libraries specific for *Burkholderia* and four general bacterial pyrosequencing libraries.

**Table 1:** **Nucleotide probes used for the PCR and FISH**.

**Name**	**Sequence (5′–3′)**	**Specificity**	**References**	**Formamide concentration (%)^a^**	**Fluorescent dye**
**PCR PRIMERS**					
Unibac-II-515f	GTGCCAGCAGCCGC	Most bacteria	Lieber et al., 2003	-	-
Unibac-II-927r	CCCGTCAATTYMTTTGAGTT	Most bacteria	Lieber et al., 2003	-	-
799f	AACMGGATTAGATACCCKG	Most bacteria	Chelius and Triplett, 2001	-	-
1492r	ACCTTGTTACGACTT	Most bacteria	Lane, 1991	-	-
BKH143Fw	TGGGGGATAGCYCGGCG	*Burkholderia* spp.	Schönmann et al., 2009	-	-
BKH1434Rw	TGCGGTTAGRCTASCYACT	*Burkholderia* spp.	Schönmann et al., 2009	-	-
**FISH PROBES**					
EUB338^b^	GCTGCCTCCCGTAGGAGT	Most bacteria	Amann et al., 1990	15	Cy3
EUB338II^b^	GCAGCCACCCGTAGGTGT	Planctomycetales	Daims et al., 1999	15	Cy3
EUB338III^b^	GCTGCCACCCGTAGGTGT	Verrucomicrobiales	Daims et al., 1999	15	Cy3
Burkho	ACCCTCTGTTCCGACCAT	*Burkholderia* spp.	Hogardt et al., 2000	40	Cy5
NONEUB	ACTCCTACGGGAGGCAGC	-	Amann et al., 1990	-^c^	-^d^

a The stringency conditions for hybridization at 41°C.

b The probes were applied together in equimolar ratio.

c The probe used for negative control at the same stringency conditions applied for positive FISH probe.

d The probe used for negative control was labeled with the same fluorescent dye as corresponding positive FISH probe.

The 16S rDNA pyrosequencing libraries were processed using the open source software package Quantitative Insights Into Microbial Ecology (QIIME) version 1.6.0 (Caporaso et al., 2010) with default parameters. The raw datasets were de-multiplexed, the primer sequences were truncated, and the datasets were filtered by removing sequences of low-quality (quality score, <25), short sequences (<200 bp), and sequences containing ambiguous characters and/or homopolymers (>6 bp). The quality-filtered datasets were de-noised and chimeras were removed if present. Sequences were clustered into operational taxonomic units (OTUs) using the UCLUST algorithm with a 97% similarity cut-off (Schloss and Handelsman, 2006; Edgar, 2010). The most abundant sequence within each OTU was taxonomically assigned using the Ribosomal Database Project (RDP) classifier with 80% confidence threshold (Wang et al., 2007). To refine the analysis, generated OTU-tables were filtered based on taxonomic metadata: OTUs classified to genera other than *Burkholderia* and OTUs containing chloroplast-derived sequences were removed from the burkholderial and general bacterial OTU-tables, correspondingly. Rarefaction analysis was performed for the complete datasets, while richness and diversity estimations were performed by calculating Chao1 and Shannon (H′) indices for the datasets normalized to the same number of sequences. For the general bacterial datasets, the occurrence of bacterial taxa was analyzed using the normalized datasets. Beta-diversity of the burkholderial datasets was analyzed using weighted UniFrac distance metric (Lozupone et al., 2010) and jackknife re-sampling (1,781 sequences per sample × 100 times). Statistical analysis was performed for the normalized datasets using the adonis test with 999 permutations (http://qiime.org/tutorials/category_comparison.html).

Representative sequences of the burkholderial OTUs were aligned with reference sequences from the non-redundant nucleotide database (nt) of the NCBI server using the BLASTN algorithm. A bootstrapped neighbor-joining phylogenetic tree of the representative sequences and the closest database matches was constructed using software packages ClustalX version 2.0.12 (Larkin et al., 2007), Phylip version 3.69 (Felsenstein, 1989), and MEGA version 4.0 (Tamura et al., 2007) as previously described (Bragina et al., 2012).

### Sequence accession numbers

The raw pyrosequencing data was deposited in the European Nucleotide Archive (ENA) under the project number PRJEB4660 with the accession numbers ERR361316–ERR361331.

### Fluorescent *in situ* hybridization and confocal laser scanning microscopy

Single plants of *S. magellanicum* and *S. fallax* were fixed with 4% paraformaldehyde/phosphate buffered salt (3:1, v/v) and stained by in-tube FISH (Grube et al., 2009). The samples were hybridized with rRNA-targeting probes (genXpress, Wiener Neudorf, Austria) specific for *Burkholderia* and with a set of universal bacterial probes. Hybridization was carried out at 41°C. The probes and corresponding stringency conditions are listed in Table 1. Confocal laser scanning microscopy (CLSM) was performed with a Leica TCS SPE confocal microscope (Leica Microsystems, Mannheim, Germany) as previously described (Bragina et al., 2012) followed by volume rendering of confocal stacks using the software Imaris 7.3 (Bitplane, Zurich, Switzerland).

## Results

### *sphagnum* mosses are preferentially colonized by burkholderial strains from the PBE cluster and a minor community fraction belongs to the plant-pathogenic cluster

**Figure 1:**
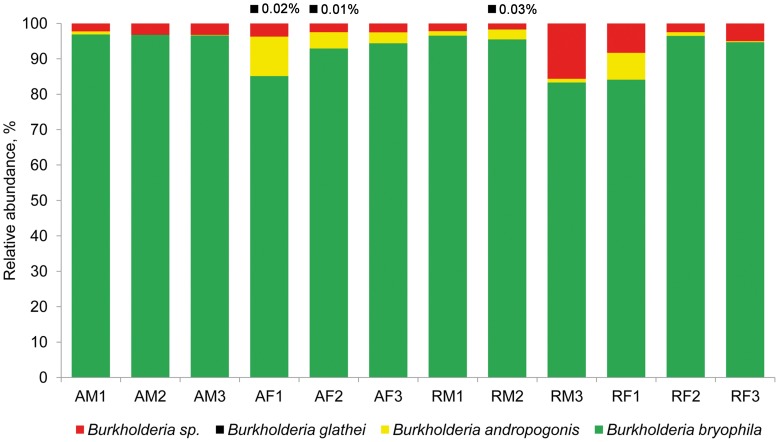
**Taxonomic classification of burkholderial communities associated with *Sphagnum* mosses.** Bar charts represent the composition of *Burkholderia*-specific 16S rDNA pyrosequencing libraries classified using RDP-classifier with a confidence threshold of 80%. The burkholderial sequences that remained unclassified at the species level are shown as *Burkholderia* sp. (red). Black squares and percentage values above the bar charts show occurrence and abundance of *B. glathei*. Abbreviations: A, Austria; R, Russia; F, *S. fallax*; M, *S. magellanicum*. Arabic numerals specify different bogs in Austria and Russia.

High-throughput analysis of the *Burkholderia* community was achieved via an amplicon pyrosequencing approach targeting the V4–V5 region of the 16S rRNA gene. The pyrosequencing of 12 amplicon libraries of *S. fallax* and *S. magellanicum* samples from Austria and Russia retrieved 149,024 raw sequences (Table 2). After initial processing, 87,917 quality sequences (average length, 405 bp) specific for *Burkholderia* genus were subjected to a detailed investigation. Rarefaction analysis of the pyrosequencing libraries, which were clustered with 97% sequence similarity, resulted in similar saturation profiles for all *Sphagnum* samples (Figure S1). Richness estimation of the normalized datasets revealed that the current pyrosequencing survey attained 81.1-100% of the estimated richness (Table 2). Low values of the Shannon diversity index (0.21–0.90, Table 2) indicated that the retrieved burkholderial communities contained a low number of highly abundant phylotypes. Through the use of automatic classification of the representative sequences, these phylotypes were assigned to *B. bryophila*, *B. andropogonis*, and *B. glathei* with several of them remaining unclassified at species level (Figure 1). According to the division of the genus *Burkholderia* sensu Suárez-Moreno et al. (2012), the most abundant *B. bryophila* species belongs to the plant-beneficial cluster, while minor *B. andropogonis* and *B. glathei* species are within the plant-pathogenic cluster.

**Table 2:** **Description and alpha-diversity estimation of the 16S rDNA pyrosequencing libraries of *Sphagnum* samples^a^**.

**Library^b^**	**Habitat**	**Country**	**Bog**	**No. of raw seq.**	**No. of filtered seq.**	**No. of OTUs (97%)**	**Chao1**	**Coverage (%)**	**Shannon, H′**
**16S rDNA LIBRARIES SPECIFIC FOR *Burkholderia*^c^**									
AM1	*S. magellanicum*	Austria	Rotmoos	12,740	9,663	3.58	3.63	98.6	0.23
AM2	*S. magellanicum*	Austria	Wasenmoos	11,750	7,393	3.57	3.72	96.0	0.21
AM3	*S. magellanicum*	Austria	Pürgschachen Moor	13,271	9,612	4.45	4.53	98.2	0.24
AF1	*S. fallax*	Austria	Rotmoos	13,189	11,016	5.64	6.25	90.2	0.75
AF2	*S. fallax*	Austria	Wasenmoos	11,987	7,870	5.18	5.37	96.5	0.46
AF3	*S. fallax*	Austria	Pürgschachen Moor	13,843	7,595	4.25	4.27	99.5	0.38
RM1	*S. magellanicum*	Russia	Polesje	12,566	9,256	4.89	4.97	98.4	0.27
RM2	*S. magellanicum*	Russia	Polewoi mys	10,213	6,632	5.04	5.38	93.8	0.31
RM3	*S. magellanicum*	Russia	Oblojni moch	12,831	2,130	5.00	5.00	100.0	0.90
RF1	*S. fallax*	Russia	Polesje	13,051	1,788	3.00	3.00	100.0	0.79
RF2	*S. fallax*	Russia	Polewoi mys	10,279	7,637	4.40	4.64	94.9	0.26
RF3	*S. fallax*	Russia	Oblojni moch	13,304	7,325	5.22	6.44	81.1	0.33
**16S rDNA LIBRARIES OF BACTERIA**									
RW	Flark water	Russia	Oblojni moch	4,296	3,934	173.00	343.26	50.4	5.65
AFG	*S. fallax* gametophyte	Austria	Pürgschachen Moor	5,399	2,869	252.00	591.94	42.6	6.47
AFS	*S. fallax* sporophyte	Austria	Rotmoos	1,665	1,051	159.00	325.14	48.9	4.93
RFS	*S. fallax* sporophyte	Russia	Polewoi mys	1,869	1,299	83.00	131.00	63.4	4.23

a Richness estimates and diversity indices were calculated for the datasets normalized to the same number of sequences per library: 1,781 (Burkholderia), 1,051 (Bacteria).

b Abbreviations specify the sampling sites and habitats: A, Austria; R, Russia; F, S. fallax; M, S. magellanicum; W, flark water; G, gametophyte; S, sporophyte. Arabic numerals specify different bogs in Austria and Russia.

c 16S rDNA pyrosequencing libraries specific for Burkholderia were obtained from gametophyte samples of Sphagnum mosses.

**Figure 2:**
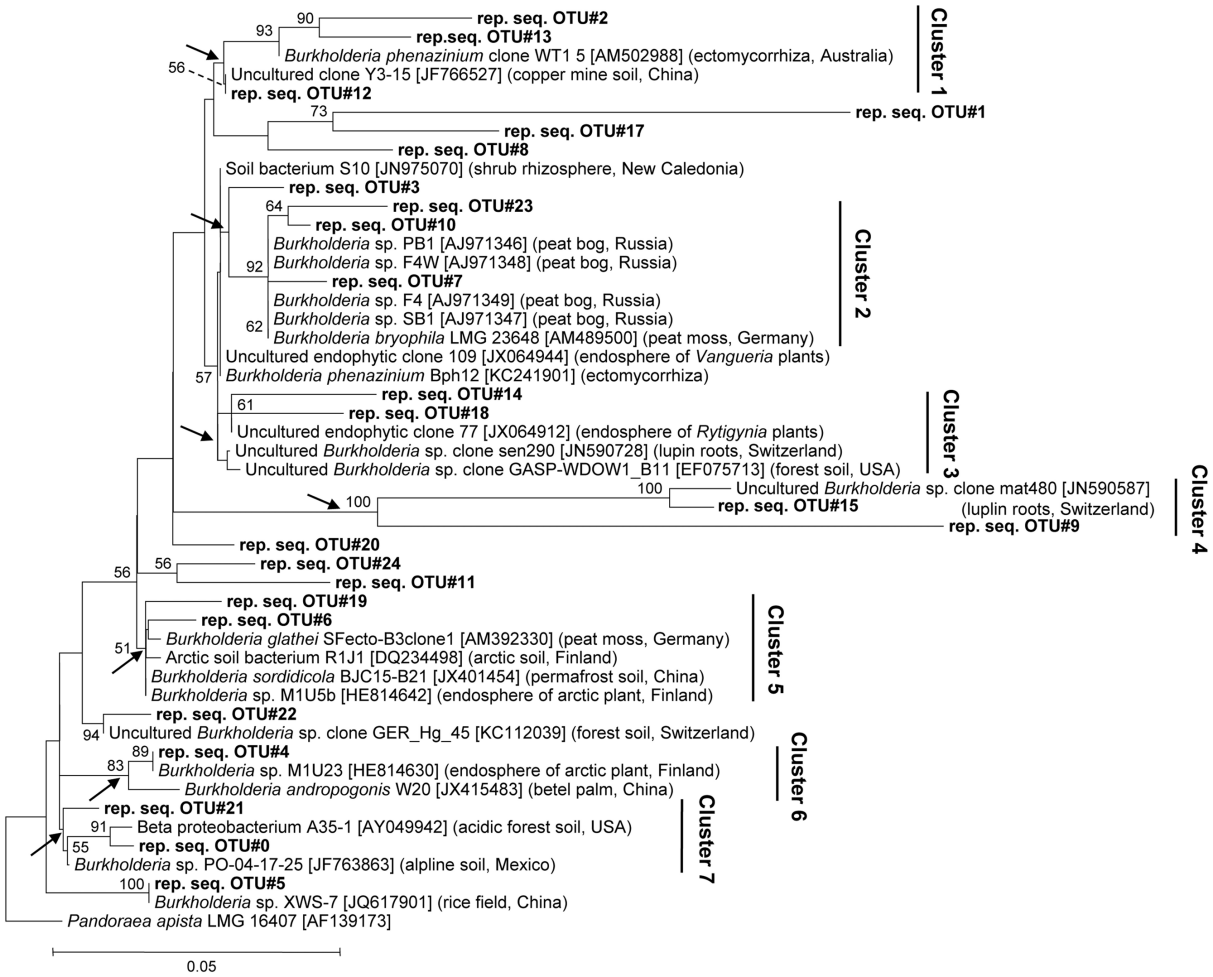
**Neighbor-joining tree of *Sphagnum*-associated burkholderial communities.** Phylogenetic relationships are shown for 16S rDNA sequence representing burkholderial OTUs (97%) (bold) and the nearest ecologically related reference sequences. The isolation sources and accession numbers of reference sequences are shown in brackets. Partial 16S rDNA sequence of *Pandoraea apista* strain LMG 16407 (AF139173) was used as an out-group. Numbers at nodes indicate bootstrap values out of 100 data re-samplings exceeding 50%. Arrows show monophyletic branches for cluster designation. Distance bar: 0.05 substitutions per site.

To achieve a deeper insight into burkholderial diversity, we performed a phylogenetic analysis of the partial 16S rRNA gene sequences from pyrosequencing libraries and closely related environmental strains (Figure 2). The closest database matches showed ≥96% of sequence identity to representative burkholderial sequences from pyrosequencing libraries. Clustering of the representative and reference sequences on the phylogenetic tree reflected several ecological traits of the examined burkholderial community. Specifically, cluster 2 was formed from representative sequences (this study) and the *B. bryophila* strain LMG 23648, a plant growth-promoting and antagonistic bacterium that was originally isolated from mosses in a nature reserve bog in Germany (Vandamme et al., 2007). This cluster also contained burkholderial strains PB1, F4W, F4, and SB1 which were isolated from acidic peat bogs in Russia (Belova et al., 2006). The phylogenetic clusters 1, 3, 4, 5, and 6 were represented by various endophytic and rhizospheric bacteria. These bacteria included the endophytic mycorrhizal *B. phenazinium* clone WT1 5, burkholderial clones sen290 and mat480 associated with lupin cluster roots (Weisskopf et al., 2011), and burkholderial endophytes M1U5b and M1U23 that were isolated from the arctic plants (Nissinen et al., 2012). Within the clusters 5 and 6, we detected *B. andropogonis* strain W20, a causative agent of the leaf spot in betel palm, and SFecto-B3clone1 clone of *B. glathei* species, a free-living or moss-associated bacterium that is not considered a member of the PBE cluster (Opelt et al., 2007a; Suárez-Moreno et al., 2012). Interestingly, the representative sequence in these clusters showed higher sequence similarity (98–100%) with the harmless burkholderial strains M1U5b and M1U23 than with *B. andropogonis* and *B. glathei* sequences. Furthermore, cluster 7 contained bacteria from acidic and alpine soils. Overall, the phylogenetic analysis revealed that *Sphagnum*-associated *Burkholderia* are phylogenetically closely related to plant-beneficial and non-pathogenic *Burkholderia* from various acidic habitats, especially peat bogs, but also potential plant pathogens.

### *burkholderia* communities of sphagna exhibit similar distribution and colonization patterns independent of the geographic region

**Figure 3:**
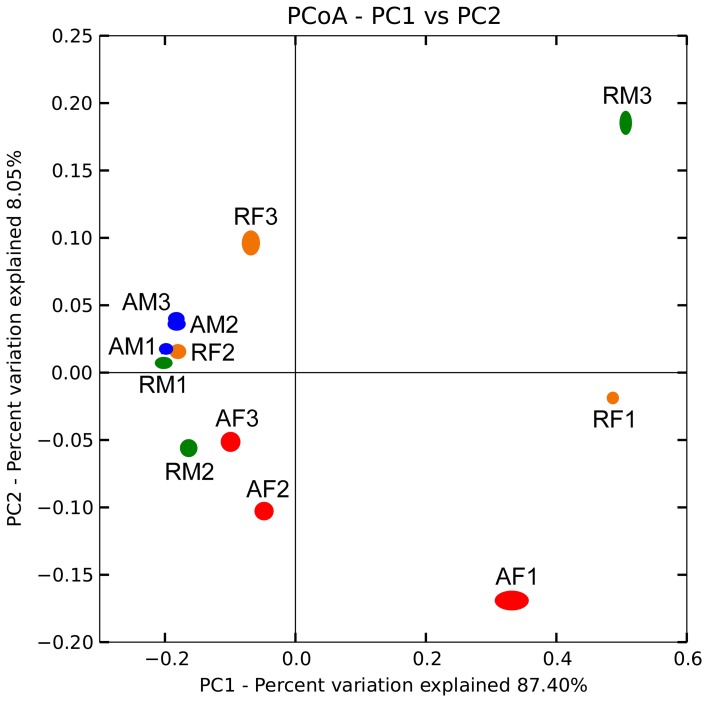
**Comparison of burkholderial communities on *Sphagnum* mosses in Austrian and Russian bogs by principal coordinate analysis (PCoA).** PCoA biplot is based on the weighted UniFrac distance matrix of the 16S rDNA pyrosequencing libraries specific for *Burkholderia* and supported by 100 jackknife data re-samplings using 1781 sequences per library. The single libraries are shown by colored ellipses: blue, *S. magellanicum* (Austria); red, *S. fallax* (Austria); green, *S. magellanicum* (Russia); orange, *S. fallax* (Russia). Letters and arabic numerals specify library names. Variation explained by each principal coordinate (PC) is defined on the biplot, respectively.

Biogeographical distribution of the *Burkholderia* communities was examined for the peat mosses *S. fallax* and *S. magellanicum* collected from Austrian and Russian bogs (Table S1). *Burkholderia* showed highly similar distribution patterns for the analyzed moss species independent of the geographic region (Figure 3). An average weighted UniFrac distance was 0.47% with a maximum value of 1.08% for *S. magellanicum*-associated communities in Russian bogs (Table S2). The statistical analysis using an adonis test confirmed that neither *Sphagnum* species (*P* = 0.261) nor geographic position (*P* = 0.363) had a significant influence on the beta-diversity of burkholderial communities.

**Figure 4:**
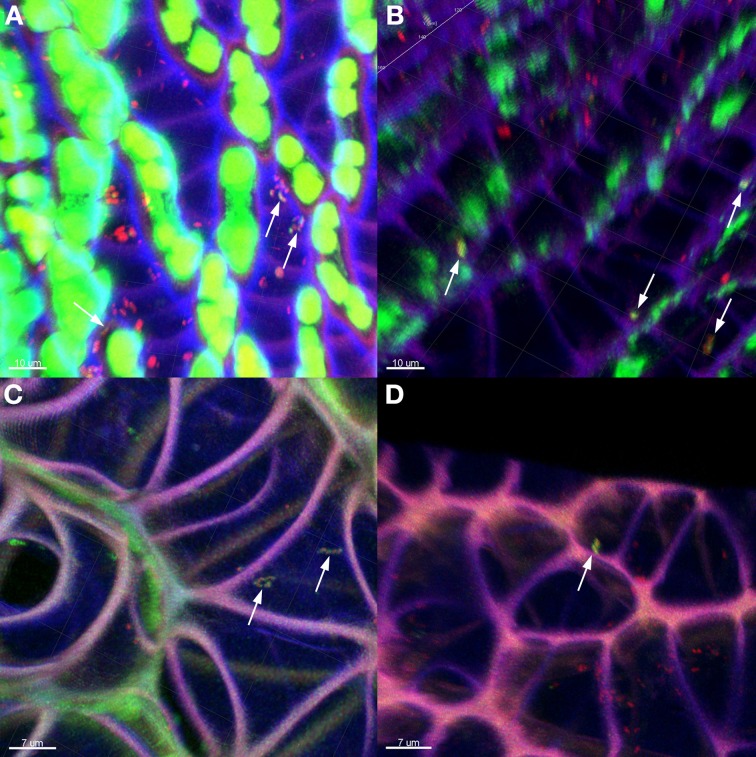
**Detection of *Burkholderia* in *Sphagnum* plants by FISH-CLSM visualization.** Branch leaves of *S. fallax* **(A,B)** and *S. magellanicum* **(C,D)** from Austrian **(A,C)** and Russian **(B,D)** bogs hybridized with *Burkholderia*-specific and universal bacterial probes. Yellow: *Burkholderia* spp. indicated by arrows; red: other bacteria; green: algae, *Sphagnum* chlorocytes; violet: moss cell walls. Scale bar = 10 μm **(A,B)**; 7 μm **(C,D)**.

The general distribution of *Burkholderia* was confirmed through fluorescent *in situ* hybridization (FISH) of *Sphagnum* gametophytes with genus-specific and universal bacterial probes (Figure 4). *Sphagnum* mosses are characterized by a unique morphology (Daniels and Eddy, 1985) which makes them easily accessible for microbial colonization (Bragina et al., 2012). CLSM observation of hybridized plants showed that the *Burkholderia* community inhabited the leaves, but not the stem tissues of mosses. Straight and slightly curved rods of *Burkholderia* were detected in hyalocyte cells of leaves being likely attached to their cell walls. Inside the hyalocytes, *Burkholderia* remained as individual cells or formed microcolonies composed of few cells. Burkholderial cells were also found in association with other bacteria of unidentified taxonomy as shown in Figure 4A. Analysis of FISH-CLSM data showed that *Burkholderia* communities established similar colonization patterns in different *Sphagnum* species across the examined geographic scales.

### *burkholderia* are vertically transmitted within the core microbiome over entire life cycle of the host plants

**Figure 5:**
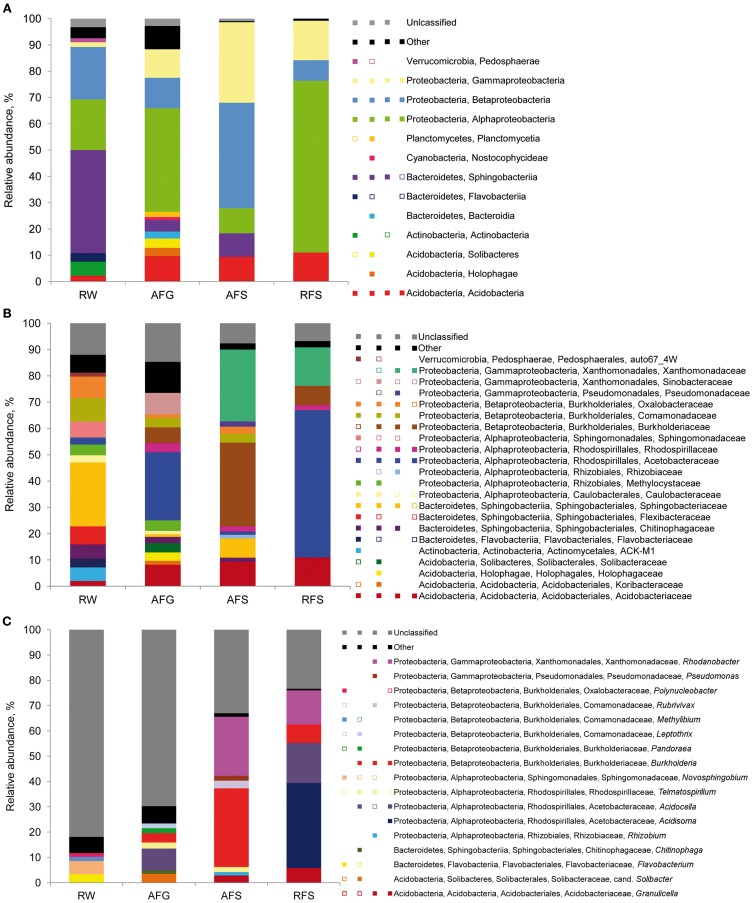
**Taxonomic classification of bacterial communities of *Sphagnum* mosses and flark water.** Bar charts represent the composition of 16S rDNA pyrosequencing libraries of Bacteria classified at class **(A)**, family **(B)** and genus **(C)** level using RDP-classifier with a confidence threshold of 80%. Multi-colored charts in the legend represent occurrence of each taxon in each library correspondingly. Taxons below 1% of relative abundance are included in ‘Other’ and depicted as empty squares on the multi-colored charts. Abbreviations: A, Austria; R, Russia; W, flark water; F, *S. fallax*; G, gametophyte, S, sporophyte.

The 16S rDNA pyrosequencing libraries from the moss sporophyte, gametophyte, and flark water samples were compared to reveal potential transmission mechanisms of *Sphagnum*-associated bacteria with a special focus on the genus *Burkholderia*. The libraries were rarefied as shown in Figure S1. The pyrosequencing survey achieved 42.6–63.4% of total richness as estimated by the Chao1 index (Table 2). Classification of the normalized datasets revealed the occurrence of certain bacterial taxa in *S. fallax* and water microhabitats (Figure 5). Thus, Proteobacteria, Bacteroidetes, and Acidobacteria were among the most abundant phyla in all examined microhabitats. At class level, Alphaproteobacteria (within the phylum Proteobacteria) comprised the dominant portion of the plant-associated microbiome, while the water microbiome was dominated by Sphingobacteria (Bacteroidetes). Furthermore, a comparison of microbiome structure at family level revealed several different occurrence patterns. For instance, *Acidobactriaceae* (within the class Acidobacteria) were ubiquitously distributed unlike the family *Xanthomonadaceae* (Gammaproteobacteria) that specifically colonized the moss-associated microhabitats, gametophyte, and sporophyte. Moreover, several bacterial taxa, namely *Methylocystaceae* (Alphaproteobacteria), inhabited moss gametophytes and flark water.

To study the occurrence of *Burkholderia* in various microhabitats, we compared the compositions of water, gametophyte-, and sporophyte-associated microbiomes at genus level. Consequently, *Burkholderia* were detected in the *Sphagnum* sporophyte and gametophyte, but were absent in the flark water. To ensure that normalization did not influence the *Burkholderia* occurrence pattern, the non-normalized pyrosequencing libraries were checked for the presence of this genus. The occurrence pattern of *Burkholderia* coincided between the normalized and non-normalized datasets (data not shown). However, pyrosequencing of the flark water microbiome achieved partial coverage of the estimated diversity (Table 2) and therefore additional experiments would be required to confirm this finding. Altogether, the obtained results indicated that the moss microbiome exhibits potential for both water-mediated and host-mediated transmission and that *Sphagnum*-associated *Burkholderia* are potentially transmitted over the entire life cycle of the host plants.

## Discussion

The genus *Burkholderia* is very important for plant and human health (Coenye and Vandamme, 2003; Suárez-Moreno et al., 2012; Mitter et al., 2013). We found that the microbiome of our model *Sphagnum* plant is preferentially enriched by the plant-beneficial and non-pathogenic *Burkholderia* from the PBE cluster, but also contains minor fraction of potential plant pathogens. We have provided new ecological insights into these important plant inhabitants including their composition, distribution, colonization, and transmission pattern.

Our hypothesis that *Sphagnum* species are colonized by specific *Burkholderia* from the PBE cluster has to be slightly revised. Although the most abundant *B. bryophila* species belongs to the plant-beneficial cluster, a minor fraction of *B. andropogonis* and *B. glathei* species are within the pathogen cluster (Suárez-Moreno et al., 2012). However, the phylogenetic analysis was crucial for elucidating their intra-specific diversity and ecological background (Figure 2). Amazingly, the resolved phylogenetic clusters contained plant-beneficial and non-pathogenic burkholderial strain as well as environmental clone sequences. This fact led us to the conclusion that *Burkholderia* members from the PBE cluster are of a great importance for the health and growth of *Sphagnum* plants. Our conclusion was supported by the isolation of *B. bryophila* and *B. phenazinium* beneficial strains from *Sphagnum* mosses at the same sampling sites by Shcherbakov et al. (2013). The minor fraction of the burkholderial community was formed by *Burkholderia* spp. from the plant-pathogenic cluster sensu Suárez-Moreno et al. (2012). However, the collected *Sphagnum* plants did not exhibit any disease symptoms. Therefore, we support the transfer of *B. glathei* the PBE cluster as recently suggested by Verstraete et al. (2013), who identified the species as common endosymbiont in plants of the *Rubiaceae* family. In contrast, *Burkholderia andropogonis* is the causal agent of numerous plant diseases affecting a wide range of monocot and dicot plants, e.g., sweet and field corn, blueberry, sorghum, carnation, coffee, statice, rye, and clover. Bacterial leaf stripe is one of the three major bacterial diseases of sorghum, and strict quarantine regulations against importation of *B. andropogonis*-infested sorghum feed grains and seeds are imposed by numerous countries (Ramundo and Claflin, 2005). *Sphagnum* mosses seem to be a natural reservoir for this plant pathogen. This is important because dry *Sphagnum* is often used for orchid and ornamental cultivation and transferred world-wide. On the other side, there are also hints that saprophytic *B. andropogonis* exists (Estrada-de los Santos et al., 2013), and many disease outbreaks depend on the abundance of pathogens and the diversity of the indigenous microbiome. At last, it is impossible to predict any pathogenic or beneficial effect from 16S rDNA analysis, and additional studies would be required to prove or contradict the pathogenicity of *Sphagnum*-associated *B. andropogonis*.

In this study, we discovered similar distribution patterns of *Sphagnum*-associated burkholderial communities independent of the geographic region, which well-confirmed our hypothesis. To elucidate this distribution pattern, we aimed to answer the question—what factors shape this community? In terrestrial habitats, pH serves as both a primary driver of microbiome structure as well as a specific determinant of the genus *Burkholderia* as it is known to exhibit pH tolerance as a general phenotypic trait (Lauber et al., 2009; Stopnisek et al., 2013). In our previous study, the same sampling sites in Austria were characterized as extremely to moderately acidic by means of Ellenberg's indicator values for pH (expressed as soil reaction) (Bragina et al., 2012). For sampling sites in Russia, the Ellenberg's values for pH varied at the same range (data not shown) and therefore all examined sites possessed favorable a-biotic conditions for the burkholderial colonization. Apart from the a-biotic factors, we previously demonstrated that various *Sphagnum* species determine the microbiome composition to different extents (Bragina et al., 2011, 2012). Through statistical analysis, we showed that neither geographic location nor *Sphagnum* species had a significant influence on the distribution of *Burkholderia*. Moreover, the similar colonization patterns of the moss-associated *Burkholderia* were verified using FISH-CLSM in a semi-quantitative way.

For a better understanding of the distribution and colonization pattern revealed for *Sphagnum*-associated *Burkholderia*, we addressed the issue of bacterial transmission in the peat bog ecosystems. Recently, Putkinen et al. (2012) described a water dispersal of methane-oxidizing bacteria in the peat bogs. Moreover, our previous study revealed that nitrogen-fixing bacteria were transferred within the moss sporophyte (Bragina et al., 2013) As a result, we hypothesized that either host-mediated or water-mediated transmission is possible for *Sphagnum*-associated *Burkholderia*. Through the comparison of microbiome composition in various bog microhabitats, we found that burkholderial communities are potentially transmitted by the host plants. The violent spore discharge and wind dispersal of the *Sphagnum* spores would enable associated bacteria to migrate over the long distances and support spore germination at a new site (Szövényi et al., 2008; Sundberg, 2010). Altogether, the detected host-mediated transmission underlines the importance of *Burkholderia* for *Sphagnum* mosses themselves and defines their distribution pattern.

In recent decades, burkholderial community was considered a typical and well-adapted component of acidic peat bogs (Belova et al., 2006). In this study, we demonstrated that *Burkholderia* associated with the main vegetation of peat bogs, *Sphagnum* mosses, contain both plant-beneficial but also potentially pathogenic *Burkholderia* that are transmitted by the host plants over their life cycle. However, global warming and human disturbance may significantly shift the environmental conditions in the peat bog ecosystems and lead to the elimination or substitution of the beneficial microbes (Dise, 2009). The obtained data supports our knowledge on native plant microbiomes and can help for the maintenance of climate-relevant bog ecosystems.

### Conflict of interest statement

The authors declare that the research was conducted in the absence of any commercial or financial relationships that could be construed as a potential conflict of interest.

## Supplementary material

Table S1 **Sampling sites**.

Table S2 **Weighted UniFrac distance matrix of 16S rDNA pyrosequencing libraries specific for *Burkholderia***.

Figure S1 **Rarefaction curves for 16S rDNA amplicon libraries of *Sphagnum* samples**.
