# Supplementary material for: Vertical transmission explains the specific Burkholderia pattern in Sphagnum mosses at multi-geographic scale
Source: Front Microbiol. 2013 Dec 18;4:394. doi: 10.3389/fmicb.2013.00394 (PMC3866706; doi:10.3389/fmicb.2013.00394)
Supplement: Table S1 — Sampling sites. [file DataSheet1.PDF]

Supplementary Material

Table S1 Sampling sites

Country, state/region	*Bog*	*Habitat*	*Sample abbreviation*[a]	*Coordinates*
Austria, Styria	Rotmoos	*S. magellanicum*	AM1rep1	N47 41.030 E15 09.276
			AM1rep2	N47 41.021 E15 09.245
			AM1rep3	N47 40.971 E15 09.270
			AM1rep4	N47 41.017 E15 09.319
		S. fallax	AF1rep1	N47 40.908 E15 09.244
			AF1rep2	N47 40.958 E15 09.175
			AF1rep3, AFS[b]	N47 41.041 E15 09.232
			AF1rep4	N47 41.055 E15 09.264
Austria, Salzburg	Wasenmoos	*S. magellanicum*	AM2rep1	N47 18.373 E12 24.927
			AM2rep2	N47 18.363 E12 24.944
			AM2rep3	N47 18.337 E12 25.119
			AM2rep4	N47 18.315 E12 25.126
		S. fallax	AF2rep1	N47 18.387 E12 24.871
			AF2rep2	N47 18.391 E12 24.866
			AF2rep3	N47 18.385 E12 24.882

			AF2rep4	N47 18.347 E12 24.980
Austria, Styria	Pürgschachen Moor	*S. magellanicum*	AM3rep1	N47 34.905 E14 20.402
			AM3rep2	N47 34.910 E14 20.454
			AM3rep3	N47 34.839 E14 20.497
			AM3rep4	N47 34.805 E14 20.493
		S. fallax	AF3rep1	N47 34.789 E14 20.398
			AF3rep2	N47 34.814 E14 20.356
			AF3rep3	N47 34.824 E14 20.346
			AF4rep4	N47 34.848 E14 20.344
Russia, Leningrad region	Polesje	*S. magellanicum*	RM1rep1	N60 44.032 E29 04.119
			RM1rep2	N60 44.024 E29 04.132
			RM1rep3	N60 44.008 E29 04.164
			RM1rep4	N60 43.994 E29 04.157
		S. fallax	RF1rep1	N60 44.044 E29 04.137
			RF1rep2	N60 44.039 E29 04.144
			RF1rep3	N60 44.027 E29 04.158
			RF1rep4	N60 44.053 E29 04.122
Russia, Leningrad region	Polewoi Mys	*S. magellanicum*	RM2rep1	N60 42.214 E29 20.803

			RM2rep2	N60 42.208 E29 20.763
			RM2rep3	N60 42.216 E29 20.818
			RM2rep4	N60 42.225 E29 20.830
		S. fallax	RF2rep1, RFS[b]	N60 42.258 E29 20.941
			RF2rep2	N60 42.256 E29 20.978
			RF2rep3	N60 42.245 E29 20.985
			RF2rep4	N60 42.234 E29 20.977
Russia, Leningrad region	Oblojni Moch	*S. magellanicum*	RM3rep1	N60 35.755 E29 08.110
			RM3rep2	N60 35.719 E29 08.083
			RM3rep3	N60 35.698 E29 08.098
			RM3rep4	N60 35.688 E29 08.123
		S. fallax	RF3rep1	N60 35.683 E29 08.188
			RF3rep2	N60 35.700 E29 08.170
			RF3rep3	N60 35.722 E29 08.133
			RF3rep4	N60 35.688 E29 08.166
		Peat water	RW	N60 35.745 E29 08.126

Abbreviations: A, Austria; R, Russia; F, *Sphagnum fallax*; M, *Sphagnum magellanicum*; S, sporophyte; W, flark water.

[a] Letters indicate countries and habitats: A, R, F, M, S, and W. Arabic numerals specify bogs and replicates.

[b] Sporophyte samples of *S. fallax* (AFS, RFS) were collected at the same sampling points as gametophyte samples AF1rep3 and RF2rep1, respectively.

Table S2 Weighted UniFrac distance matrix[a] of 16S rDNA pyrosequencing libraries specific for *Burkholderia*[b].

	AM1[c]	AM2	AM3	AF1	AF2	AF3	RM1	RM2	RM3	RF1	RF2	RF3
AM1	0.00	0.07	0.08	0.84	0.29	0.19	0.06	0.13	1.07	1.01	0.09	0.29
AM2	0.07	0.00	0.05	0.80	0.34	0.20	0.11	0.17	1.03	0.97	0.06	0.23
AM3	0.08	0.05	0.00	0.80	0.31	0.17	0.10	0.15	1.01	0.97	0.05	0.23
AF1	0.84	0.80	0.80	0.00	0.57	0.67	0.84	0.79	0.56	0.29	0.75	0.72
AF2	0.29	0.34	0.31	0.57	0.00	0.14	0.30	0.24	0.91	0.74	0.28	0.30
AF3	0.19	0.20	0.17	0.67	0.14	0.00	0.19	0.13	0.94	0.84	0.14	0.18
RM1	0.06	0.11	0.10	0.84	0.30	0.19	0.00	0.09	1.08	1.01	0.10	0.30
RM2	0.13	0.17	0.15	0.79	0.24	0.13	0.09	0.00	1.07	0.96	0.11	0.28
RM3	1.07	1.03	1.01	0.56	0.91	0.94	1.08	1.07	0.00	0.27	0.98	0.83
RF1	1.01	0.97	0.97	0.29	0.74	0.84	1.01	0.96	0.27	0.00	0.92	0.77
RF2	0.09	0.06	0.05	0.75	0.28	0.14	0.10	0.11	0.98	0.92	0.00	0.20
RF3	0.29	0.23	0.23	0.72	0.30	0.18	0.30	0.28	0.83	0.77	0.20	0.00

Abbreviations: A, Austria; R, Russia; F, *Sphagnum fallax*; M, *Sphagnum magellanicum*.

[a] Weighted UniFrac distance metric is expressed in percent.

[b] Pyrosequencing libraries were normalized to the same number of sequences per library (1781).

[c] Letters indicate countries and *Sphagnum* species: A, R, F, M. Arabic numerals specify different bogs in Austria and Russia.

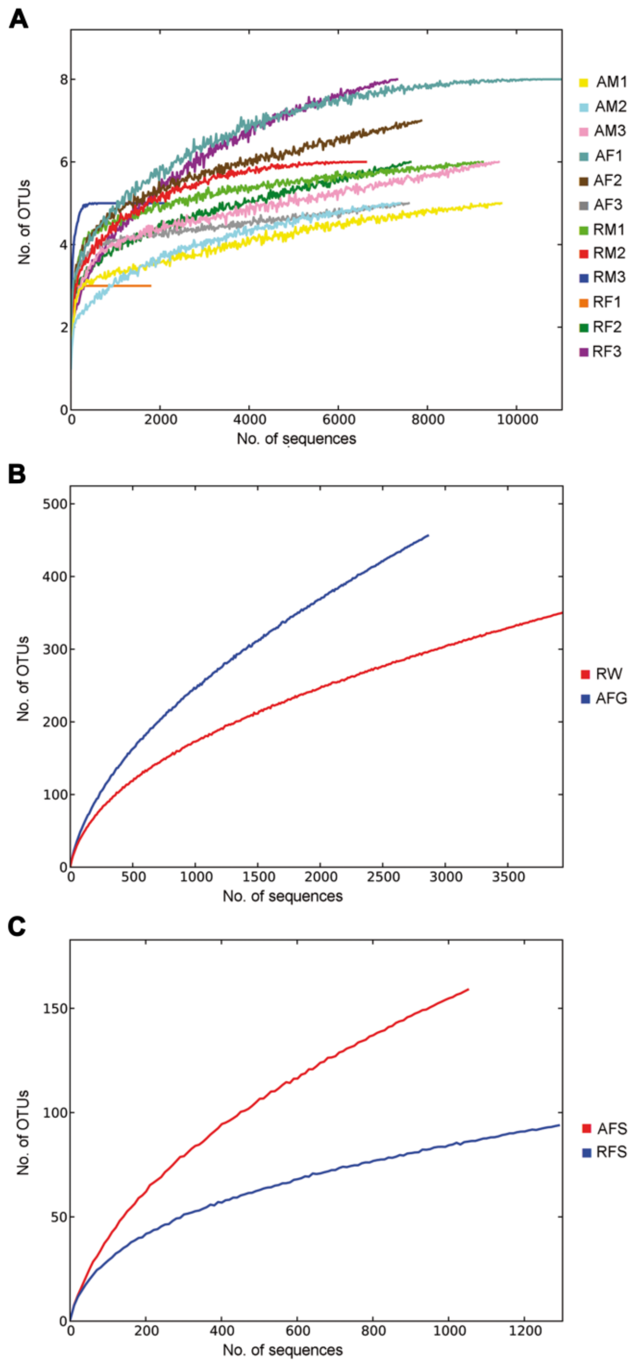

Figure S1 Rarefaction curves for 16S rDNA amplicon libraries of *Sphagnum* samples. Rarefaction curves represent richness of the *Burkholderia*-specific (**A**) and general bacterial (**B**, **C**) datasets clustered with 97% similarity cut-off. Samples are abbreviated as follows: A, Austria; R, Russia; F, *S. fallax*; M, *S. magellanicum*; W, flark water; G, gametophyte; S, sporophyte. Arabic numerals specify different bogs in Austria and Russia.
